# Towards Interpretable Machine Learning for Automated Damage Detection Based on Ultrasonic Guided Waves

**DOI:** 10.3390/s22010406

**Published:** 2022-01-05

**Authors:** Christopher Schnur, Payman Goodarzi, Yevgeniya Lugovtsova, Jannis Bulling, Jens Prager, Kilian Tschöke, Jochen Moll, Andreas Schütze, Tizian Schneider

**Affiliations:** 1 Lab for Measurement Technology, Saarland University, 66123 Saarbrücken, Germany; p.goodarzi@lmt.uni-saarland.de (P.G.); schuetze@lmt.uni-saarland.de (A.S.); t.schneider@lmt.uni-saarland.de (T.S.); 2 Department of Non-Destructive Testing, Acoustic and Electromagnetic Methods Division, Bundesanstalt für Materialforschung und -Prüfung (BAM), 12205 Berlin, Germany; yevgeniya.lugovtsova@bam.de (Y.L.); jannis.bulling@bam.de (J.B.); jens.prager@bam.de (J.P.); 3 Systems for Condition Monitoring, Fraunhofer Institute for Ceramic Technologies and Systems IKTS, 01109 Dresden, Germany; kilian.tschoeke@ikts.fraunhofer.de; 4 Department of Physics, Goethe University Frankfurt, 60438 Frankfurt, Germany; moll@physik.uni-frankfurt.de; 5 Research Group Data Engineering and Smart Sensors, ZeMA—Center for Mechatronics and Automation Technology gGmbH, 66121 Saarbrücken, Germany

**Keywords:** composite structures, structural health monitoring, carbon fibre-reinforced plastic, interpretable machine learning, automotive industry

## Abstract

Data-driven analysis for damage assessment has a large potential in structural health monitoring (SHM) systems, where sensors are permanently attached to the structure, enabling continuous and frequent measurements. In this contribution, we propose a machine learning (ML) approach for automated damage detection, based on an ML toolbox for industrial condition monitoring. The toolbox combines multiple complementary algorithms for feature extraction and selection and automatically chooses the best combination of methods for the dataset at hand. Here, this toolbox is applied to a guided wave-based SHM dataset for varying temperatures and damage locations, which is freely available on the Open Guided Waves platform. A classification rate of 96.2% is achieved, demonstrating reliable and automated damage detection. Moreover, the ability of the ML model to identify a damaged structure at untrained damage locations and temperatures is demonstrated.

## 1. Introduction

Machine learning (ML) techniques require a large number of measurements for adequate training and reliable decision-making. Therefore, ML is well suited for structural health monitoring (SHM) applications in which one or multiple sensors are permanently attached to the structure so that structural measurements can be recorded frequently. This rich data pool can be exploited by ML techniques to train a model that can detect damages or anomalies, allowing for fully automated damage detection.

Several ML methods have been developed in the last few years to solve various SHM and damage detection problems, especially by using neural networks (NN) [1,2,3,4,5]. Even though ML methods are already well established in vibration-based SHM [6], their use in guided wave-based SHM is currently rising [7,8,9]. For instance, Roy et al. [7] described an unsupervised learning approach for structural damage identification under varying temperatures based on an NN. Their methodology is validated with measurements from coupon samples in a uniaxial testing machine. More recently, Miorelli et al. [8] demonstrated that support vector machines (SVM) trained on numerical data can be used to solve the inverse problem for damage detection and sizing from experimental guided wave (GW) images. They used a circular array of transducers on an isotropic metal plate with through-holes of different sizes modelled at different locations. Mariani et al. [9] showed improvements in automatic damage detection when using a causal dilated convolutional NN without the need for feature engineering by a human operator. Qiu [1] studied Gaussian mixture models for GW in SHM systems using measurements from a full-scale fatigue test. 

Keogh et al. [10] found, in a study of 340 papers, that new methods are tested on average on 1.3 different datasets and compared to 0.9 other methods only, and routine applications are desired to reduce the requirement for data scientists to adapt the ML methods for industrial applications. The contribution of this work is therefore the adaptation and application of an existing ML framework previously used for condition monitoring of industrial machines to GW-based SHM to enable autonomous damage detection. The framework is based on a toolbox combining multiple established ML algorithms that was successfully applied to various other datasets (cf. [11]). A reference dataset from the Open Guided Waves platform is used in this work, consisting of GW measurements performed with an array of piezoelectric transducers on a carbon fibre-reinforced polymer (CFRP) under varying temperatures [12]. The analysis shows that a classification rate of 96.2% can be achieved, demonstrating reliable and automated damage detection. Moreover, the ability of the ML model to detect damages at untrained damage locations and temperatures outside of the trained temperature range is also demonstrated. The methodology presented in this manuscript should be seen as a general pathfinder rather than a tailored solution.

Neural networks are commonly used in SHM applications but are difficult to interpret, and therefore their use in safety-relevant applications is limited. The methodology presented in this paper focuses on interpretability, meaning that the ML results must be physically interpretable to enable the use of ML also in safety-relevant applications. We compare our methodology against the performance of an NN applied on the same dataset [9]. Furthermore, we demonstrate that our ML methodology enables a straightforward learning procedure without the need for domain-specific knowledge and highly educated staff like data scientists, which is very important for wider application of these methods in the industry. On the other hand, it must be noted that even better performance can be achieved with domain-specific knowledge by highly educated staff.

The outline of the paper is as follows. First, the experimental setup along with the pre-processing of signals for temperature compensation is presented, followed by the description of the automated toolbox. Next, the performance of the automated ML framework is analysed. To do so, a realistic validation scenario is chosen, which is a crucial step to minimise overfitting. In addition, the selection of the hyper-parameters is motivated to achieve a higher performance. The Results section first provides a visualisation of the data using principal component analysis. Then, the performance of different algorithms for automated damage detection is presented and discussed. Moreover, the robustness of the algorithms against different damage locations and temperatures is tested and a comparison to results achieved with a deep learning NN by Mariani et al. [9] is presented. The paper closes with conclusions and the outlook.

## 2. Machine Learning Approach

### 2.1. Description of the Experimental Setup

This study is based on a freely available benchmark dataset for guided wave-based SHM with varying temperatures, recorded by Moll et al. [12]. Here, multiple ultrasonic transducers (T_1_–T_12_) were attached to a carbon fibre-reinforced plastic (CFRP) plate, as well as a sequentially added detachable mass (aluminium disc) at four different locations (D_04_, D_12_, D_16_, D_24_) to simulate structural damages. The impact of the simulated damages on the measurements can be considered a rough approximation of real delamination (e.g., decrease in amplitude and changes in time of flight) [12]. The exact positions of the transducers and the damage locations as well as their distance to the direct signal path (T_4_ to T_9_) can be found in Table 1. Note that, in the scope of this manuscript, the term “simulated damage” denotes an experimental simulation of a damaged material and does not refer to numerical simulation.

**Figure 1:**
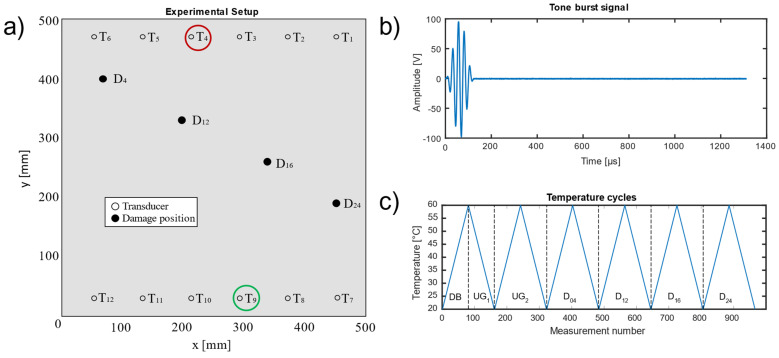
(**a**) Schematic of the experimental setup [12]. The analysed sensor combination is indicated by circles (the red circle indicates the transmitter T4, whereas the green circle indicates the receiver T9). The considered damage positions (D_04_, D_12_, D_16_, D_24_) are indicated by filled black dots. (**b**) 40 kHz Hann-windowed tone-burst signal with five cycles. (**c**) Temperature of the climatic chamber for each measurement number, where the dotted lines indicate the corresponding groups of the database, undamaged and damaged measurements.

A schematic of the CFRP plate with the positions of the transducers and damages is shown in Figure 1a. The subsequent analysis considers the case of a 40 kHz Hann-windowed tone-burst signal with five cycles (Figure 1b) sent by T_4_ and received by T_9_ for all four damage locations D_04_, D_12_, D_16_, and D_24_ as well the undamaged structure. Each measurement contains only one simulated damage at a time. During the experiment, the plate was subjected to several temperature cycles between 20 and 60 °C in a climatic chamber (Figure 1c) at constant humidity (50% RH, mean: ~50.1%, standard deviation ~0.3%). For studies concerning the impact of humidity on CFRP the reader is referred to Schubert et al. [13]. Note that measurements for the undamaged plate were performed on two temperature cycles instead of only one. For the pre-processing (Section 2.2) the ascending flank (20 °C to 60 °C in 0.5 °C steps) of the first temperature cycle of the undamaged plate was used as a database (DB, Figure 1c) for the optimal baseline selection (OBS) of reference signals (cf. Section 2.2), and the descending flank is labelled “undamaged group 1” (UG_1_). The second temperature cycle (ascending and descending flank) is labelled “undamaged group 2” (UG_2_). These two different groups are later used in the validation (Section 2.4). 

Multiple configurations were analysed and two representative scenarios chosen, one where the transducers were located in the middle of the CFRP plate (T_4_ and T_9_) and the other where they were located at the edge (T_1_ and T_7_; Section 3.3). In the scope of this study, we focused on one transducer combination at a time to be able to interpret the ML results more easily and, more importantly, to reduce the complexity and cost of later SHM configurations. Although the performance could be increased by using the information of all sensors, the aim of this study was to gain a better understanding of which configuration is necessary to reliably detect a damaged structure. 

### 2.2. Signal Pre-Processing

**Figure 2:**
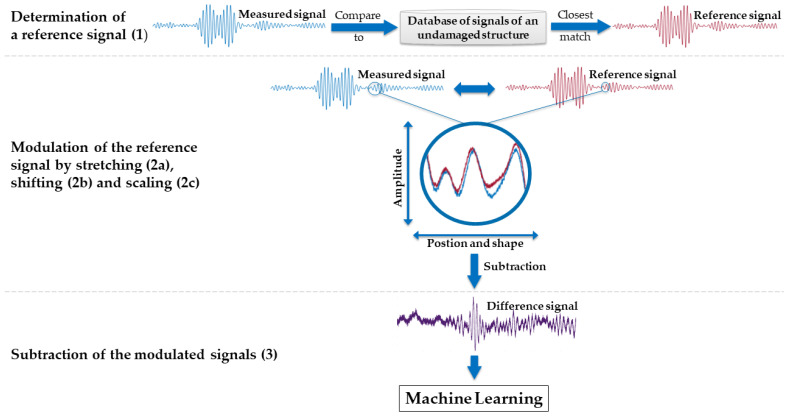
Pre-processing of the raw data to compensate for temperature-related effects by using optimal baseline selection (OBS) and baseline signal stretch (BSS).

**Figure A1:**
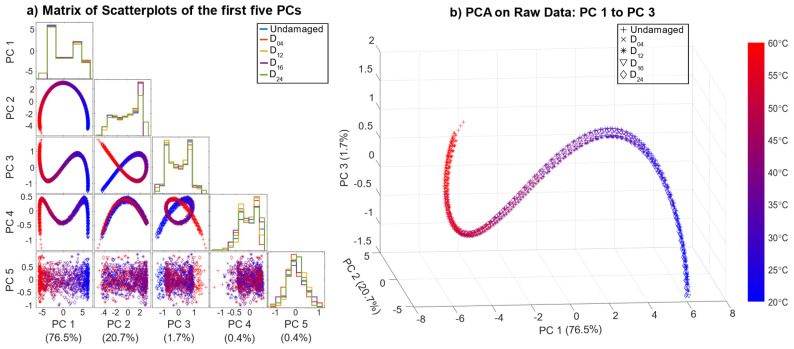
(**a**) Matrix of the first five PCs of the PCA on the raw data (undamaged plate and all simulated damage locations) coloured by their corresponding temperature with their histograms on the diagonal and the variance explained by each PC given as percentage in brackets. (**b**) First three PCs plotted into a three-dimensional space.

Increasing the temperature of the CFRP decreases the phase and group velocity of guided wave modes and increases material attenuation. Unsupervised principal component analysis (PCA) on the raw data identifies this effect to be by far the most dominant variation in the dataset (Appendix A, Figure A1). It masks less significant fault symptoms that indicate a damage in the CFRP specimen. This may cause the unsupervised and automated feature extraction strategy described below to miss these symptoms. To mitigate this effect, differential measurement techniques—optimal baseline selection (OBS) and baseline signal stretch (BSS)—were employed for temperature compensation [14]. This approach is schematically shown in Figure 2 and comprises the following steps:

OBS is applied, where the measured signal is compared to all signals of the reference database from the intact structure covering the full experimental temperature range. The closest match (reference signal) as determined by the root mean square error (RMSE) is chosen as the optimal baseline.

BSS is applied on the baseline signal:

- The baseline signal is stretched on the time axis to best fit the measured signal, again as determined by the RMSE.
- The stretched baseline is shifted on the time axis to achieve the best fit to the measured signal in terms of RMSE.
- The shifted baseline’s amplitude is scaled to match the measured signal in terms of RMSE.

This modified baseline is subtracted from the measured signal to obtain the difference (residual) signal. 

All approaches, methods, and results reported below are based on the signals taken from the reference database being pre-processed using OBS and BSS algorithms.

The database in this study contained 81 measurements with only one measurement per 0.5 °C temperature step (cf. Section 2.1). Here, we selected the minimum database that contained all temperatures to keep the computation time low, since OBS compares measured signals to each signal in the database. In real-life SHM applications, the number of measurements of an intact structure could be much higher by adding every new measurement (of an intact structure) to the database, rapidly increasing its size. However, we suggest focussing on the composition of the database rather than its size because a database representing a high variance of, e.g., environmental conditions like temperature, humidity, etc., should increase the robustness of the ML model.

### 2.3. Automated Toolbox

Signal classification was performed using a fully automated toolbox for industrial time series feature extraction and selection [15]. All algorithms are part of the MATLAB-based open-source Automated ML Toolbox for Cyclic Sensor Data [16] and its compiled version DAV³E—Data Analysis and Verification/Visualisation/Validation Environment [17] (Supplementary Materials), both developed by the Lab for Measurement Technology at Saarland University. This automated toolbox combines five unsupervised and complementary feature extraction (FE) methods with three complementary methods for feature selection (FS) (Table 2).

To keep the computation within a reasonable time, the extracted number of features was reduced in a first feature (pre-)selection to the 500 features with the highest PCC. Thus, 15 FE/FS combinations were automatically analysed within the toolbox, using a simple classification approach based on supervised linear discriminant analysis (LDA) with Mahalanobis distance classification [28]. Out of the 15 combinations, the best FE/FS combination was automatically selected based on the highest test accuracy using 10-fold cross-validation.

If needed, this approach can be extended using more sophisticated classification algorithms. In this study, further investigations with a support vector machine (SVM) with a radial basis function kernel (RBF-Kernel) were performed, because this classifier achieved the best performance (highest accuracy in the shortest time) in a comparison of 14 different families of classification algorithms on 115 binary datasets [29]. Other relevant examples of using SVM in the context of SHM can be found in [6,8].

### 2.4. Validation Scenario

**Figure 3:**
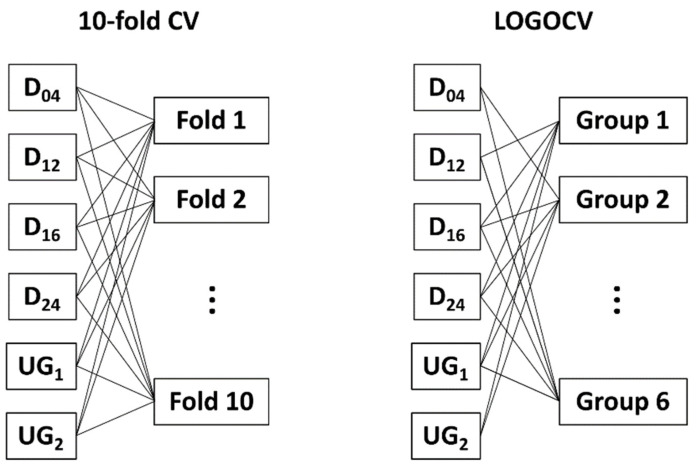
Comparison of 10-fold CV (**left**) and LOGOCV (**right**).

In real-world applications, the exact position of damage is unknown and generally differs from simulated or trained ones. Therefore, damage detection is required to also detect damages located at positions that were not included in the training data by learning certain global damage characteristics that are robust against changes in damage location. Thus, the model is trained with the pre-processed data as a binary decision (damaged/undamaged). The standard stratified 10-fold cross-validation (Figure 3, left) divides the dataset into 10 sub-datasets (folds), where each fold has the same proportion of damaged and undamaged data. Here, simple ML approaches can achieve a high accuracy on the Open Guided Wave data, which shows statistical significance but not the needed robustness against untrained damage positions, since all simulated damages (D_04_, D_12_, D_16_, D_24_) are included in each training set. Stratified CV cannot guarantee that the model learns general characteristics of a damaged or undamaged structure instead of only damage-specific and position-related characteristics, which only occur at the locations of the trained damages. This may result in overfitting, meaning that the ML model is trained only for specific damage locations and is then unable to identify damages at other locations. Therefore, 10-fold cross-validation is replaced by leave-one-group-out cross-validation (LOGOCV; Figure 3, right). To do so, the dataset is divided into data subsets with respect to the corresponding groups (UG_1_, UG_2_, D_04_, D_12_, D_16_, D_24_), allowing for the exclusion of each damage location from the training data once and thus making this damage location completely unknown to the ML model. The excluded group is then used to validate the performance of the trained model. To ensure that the training dataset always contains data of the undamaged sample, these measurements are split into two groups (UG_1,_ UG_2_). 

**Figure 4:**
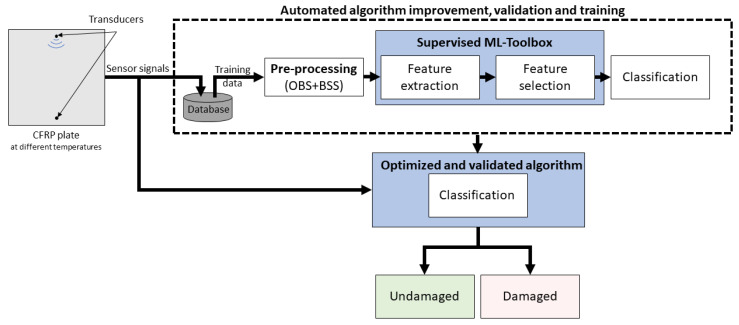
Flowchart illustrating the ML framework and the decision-making strategy.

The flowchart of this methodology is depicted in Figure 4. It shows how the sensor signals are used for the training and automated algorithm selection. After selecting the best FE method in combination with the chosen robust feature selection (RELIEFF) and classification (SVM with RBF kernel) based on testing with LOGOCV, the model is trained with all available data. It is then applied to new measurements, classifying them as either damaged or undamaged.

### 2.5. Hyper-Parameter Selection

To increase the performance of the ML model, a selection of the hyper-parameters *C* (regularisation parameter of the SVM) and the number of features was performed. Here, a grid search approach was used based on Gui et al., who tested three methods for SVM optimisation in SHM for damage detection with a grid search, achieving the highest accuracy [30]. In this approach, an ML model is trained and validated with every possible combination of hyper-parameters in a pre-defined range. The combination with the highest validation accuracy is chosen and finally tested with independent data not included in the training and validation data. 

Table 3 shows the values and tested number of values for each parameter. To reduce computational time and resources while still covering a broad range of values, the step size for the number of features increased the higher it became. The maximum number of features was set to 500 based on the feature pre-selection, which reduced the number of extracted features to 500 to avoid overfitting. Similarly, to cover a wide range of values for the regularisation parameter C, logarithmic scaling was chosen, i.e., $C=10^{0.5i},\;i\;\epsilon \;\left(-2,8\right)$.

Note that the parameter σ of Equation (A5) (cf. Appendix B) was not part of the grid search, as it is automatically optimised by MATLAB. After performing the grid search approach, the algorithm selects a parameter combination achieving high accuracy while using as few features as possible. Regarding the regularisation parameter C, if multiple parameter combinations achieve maximum accuracy, a trade-off can be made. Whereas a larger value for C suppresses misclassifications, a smaller value for C allows misclassifications to a certain degree [31]. Here, we preferred a smaller value for C to achieve a higher tolerance for misclassifications and higher robustness against outliers [31]. Further information on the theoretical background of SVMs can be found in [31,32] on the difference between hyper-parameter tuning as performed here and hyper-parameter optimisation of SVMs as described in [33,34,35].

## 3. Results and Discussion

### 3.1. Principle Component Analysis

**Figure 5:**
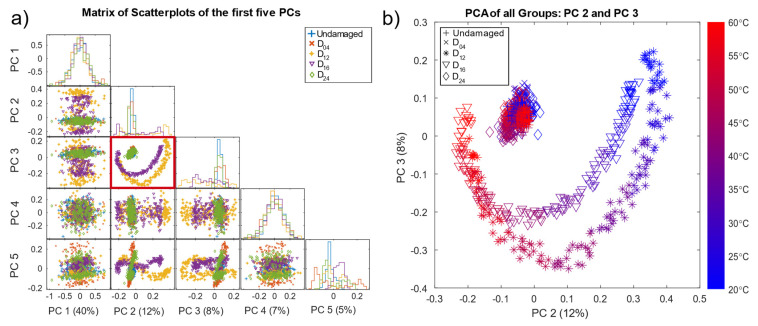
(**a**) Matrix of the first five PCs of the PCA on the pre-processed data (undamaged plate and all simulated damage locations) with their histograms on the diagonal and the variance explained by each PC given as a percentage in brackets. The red box indicating the scatterplot of PC 2 and PC 3 is also shown in (**b**), where the data points are additionally coloured by their corresponding temperature.

Principal component analysis is a common unsupervised method for visualising data to gain a better understanding of the nature of the dataset. Figure 5a shows the result of the scatterplots of the first five principal components (PC) based on the pre-processed data, with the corresponding variance that each principal component explains and the histograms on the diagonal. Here, the second and third PC (PC2, PC3), indicated by a red box, showed better separability than the remaining PCs. Note that PCA is used here for visualisation of the pre-processed data (OBS + BSS) only, without any additional data treatment.

The scatter plot of PC 2 and PC 3 (Figure 5b) reveals good separability for damage locations D_12_ and D_16_ located in the direct signal path between T4 and T9, where waves reflected from and transmitted through the damage (resulting in decreased amplitudes) had a higher impact on the measurements. Since D_04_ and D_24_ were not in the direct signal path, their influence on the received signal was smaller. D_04_, D_24_, and the undamaged data formed a cluster in the centre. In addition, Figure 5b shows all pre-processed measurements coloured by the corresponding temperature. Thus, the crescent-moon shape of the signals for D_12_ and D_16_ was mainly due to the temperature effect, which was not fully compensated by the OBS + BSS pre-processing. Figure 5b implies that measurements of D_12_ and D_16_ at higher temperatures were more difficult to discriminate, as they lay closer to each other as well as to the cluster of the undamaged plate and damages D_04_ and D_24_.

These plots also show that pre-processing can, at least to a certain degree, suppress temperature effects and highlight damage symptoms. However, the damage cases D_04_ and D_24_ overlapped with the undamaged data UG_1_ and UG_2_ in the first five PCs, which explains 72% of the variance. 

### 3.2. Results of the Automated Toolbox and Improvement of the Algorithms

In the following, we describe our approach to find a robust model with a high classification rate. When using the standard classifier of the toolbox, the highest resulting test accuracy was 88%, achieved using BFC as a feature extractor and RFE-SVM for feature selection (Table 4). This classification rate is inadequate, especially for safety-relevant applications. Table 4 provides further information on how the different FE/FS combinations performed. Here, a user of the toolbox could see that, besides the expected BFC extractor, the SM extractor might be interesting for further analysis, whereas, e.g., ALA is not suitable for FE here.

To increase the performance, the feature extraction method was improved, and the feature selection and classification methods were replaced. Due to the relatively high robustness against incomplete and noisy data in real-life applications, RELIEFF was chosen as the feature selection algorithm [25,26]. As a classifier, SVM with RBF kernel was chosen due to its good performance in a comparison of 14 families of classification algorithms on 115 binary datasets [19].

The BFC extractor of the toolbox initially extracted 5% (1310 features) of the frequency spectrum by ranking them according to the highest amplitude, and extracted those frequencies and their corresponding phase angles. This value was increased up to 10% (2620 features) to also consider features with a lower signal amplitude in the training. To achieve a reasonable computing time, the resulting 2620 features were first reduced to 500 by selecting the features with the highest Pearson correlation to the damage. The final FS method, RELIEFF, reduced the number of features down to 20. This number of features was determined by averaging the obtained feature numbers of the six models in the grid search. This improvement of the toolbox resulted in a damage classification rate of 96.2% (Table 5) compared to 88%, i.e., reducing the number of misclassified measurements from 118 to 33. A detailed description of the improved algorithms and the procedure is given in Appendix B.

**Figure 6:**
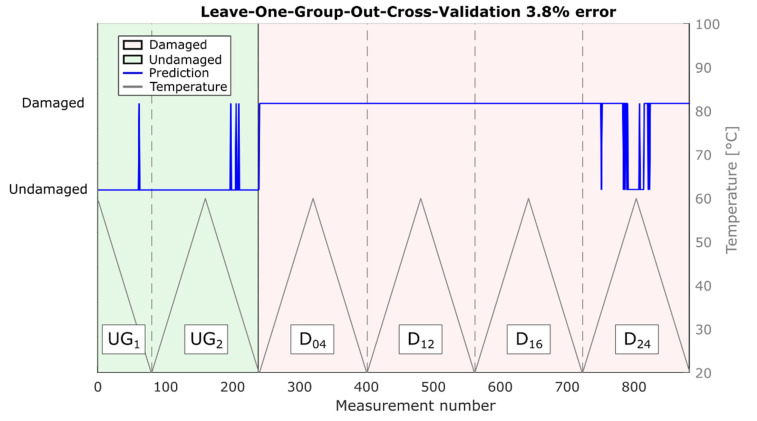
Damage classification results of the leave-one-class-out cross-validation. The plot is divided into six sections by dotted lines. Each section represents a heat cycle with one specific damage condition (undamaged and damaged D_04_, D_12_, D_16_, D_24_).

It is worth mentioning that due to the validation strategy (LOGOCV), these results are robust for temperature variations as well as damages at unknown positions. The corresponding predictions are shown in Figure 6. Note that most misclassifications occurred for measurements of damage at position D_24_, which is the location farthest from the direct path in this study (186 mm; Table 1), in combination with high temperatures (>45 °C).

With the proposed transparent FE/FS approach, the ranking of the features that are most often selected for damage detection can help with a physical interpretation. The five highest ranks (eight features) are listed in Table 6.

**Figure 7:**
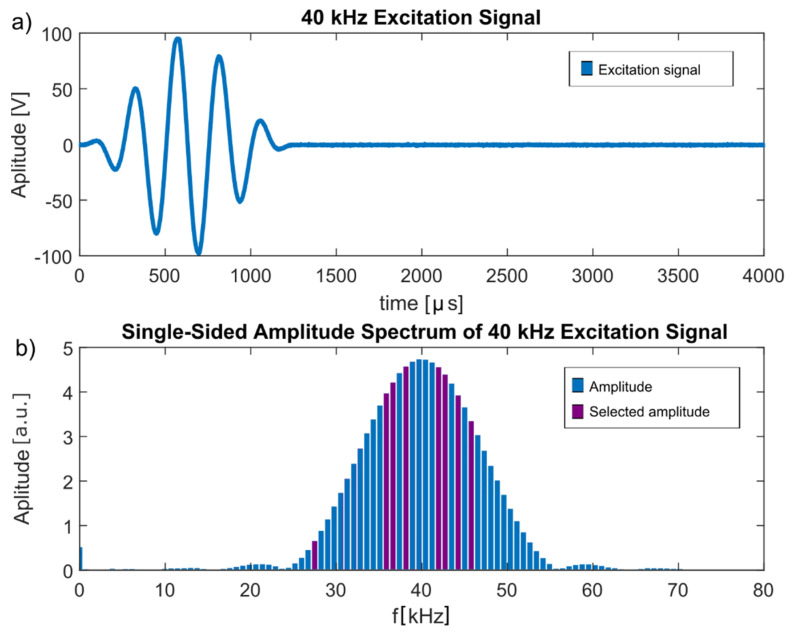
(**a**) 40 kHz excitation signal. (**b**) Single-sided amplitude spectrum of the 40 kHz excitation signal. Purple bars indicate the frequencies selected by the improved algorithm of the automated toolbox.

These frequencies were all included in the frequency spectrum of the Hann-windowed excitation frequency, as shown in Figure 7, indicating that they were not a misinterpretation of environmental influences but indeed originated from the excitation signal.

### 3.3. Influence of the Distance between Damage Location and Signal Path

Incorrectly classified data samples resulted mostly from signals of damage location D_24_, which required a considerable extrapolation since this damage location was furthest from the signal path (186 mm; Table 1), which is believed to have had a significant influence on the ML performance, especially at higher temperatures. Therefore, we performed an additional investigation of the combination of transducers 1 and 7 (Table 7), where D_24_ lay in the direct signal path. Table 8 shows the distances of each damage location from the direct signal path for this transducer combination.

The results given in Table 9 show the same tendency as for the combination of transducers 4 and 9: D_24_ and D_16_ were close to the signal path; thus, they were classified correctly, whereas the accuracy dropped with increasing distance between damage location and signal path. The reduced accuracies for the undamaged cases (UG_1_, UG_2_) were possibly due to features present in the damage cases being similar to features of the undamaged case; however, this needs to be investigated further.

### 3.4. Robustness against Temperature Influences

**Figure 8:**
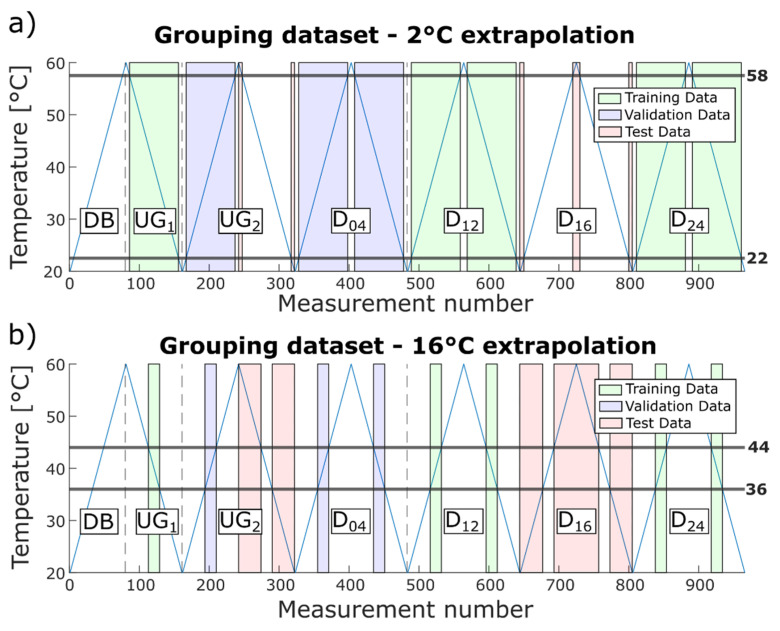
Grouping of the data into training, validation, and test data for (**a**) 2 °C and (**b**) 16 °C extrapolation.

The temperature range tested by Moll et al. [12] simulates conditions from room temperature up to 60 °C in 0.5 °C steps, making it suitable primarily for indoor applications, e.g., lightweight manipulators for robots [37]. To also cover outdoor applications, e.g., rotor blades of wind turbines, which have to withstand temperatures in the range from −50 °C to +100 °C [38], the temperature range needs to be extended in future experiments. To investigate the influence of a smaller temperature range while training the ML model, i.e., to check how well the model can extrapolate, a training temperature range was successively reduced, extending the required extrapolation from 2 °C to 16 °C in 2 °C steps. In the scope of this manuscript, extrapolation denotes testing of measurements that were performed outside the trained temperature range. Thus, a model was first built using the temperature range 22.5 °C to 57.5 °C for training and validation, then it was tested for the temperature ranges 20 °C to 22 °C and 58 °C to 60 °C, and then further the training range was further reduced and the test temperature range increased. Within each case, data from UG_1_, D_12_, and D_24_ were used for training, and data from D_04_ and the rising temperature flank of UG_2_ for validation. The extended temperature range of these data plus the respective data from D_16_ and the descending flank of UG_2_ were used for testing, as shown in Figure 8a,b for 2 °C and 16 °C extrapolation, respectively.

Note that further extrapolation is not meaningful since the size of the training data set was reduced with every step, decreasing the statistical significance. For 16 °C extrapolation, the training data (green areas in Figure 8b) only contained 75 measurements in the range of 36.5 °C to 43.5 °C. 

Table 10 shows the test accuracies achieved for each temperature extrapolation step. The ML model extrapolated up to 6 °C without loss of performance and had only a slight decrease in performance for temperature extrapolations up to 10 °C, indicating that the model is fairly robust to temperature influences. This might allow a model to be built based on data from a lab environment that could still achieve acceptable performance under real operating conditions. Note that extrapolation over 12 °C corresponds to a training range from 32.5 °C to 47.5 °C, i.e., ΔT = 15 °C. Thus, only approx. one third of the overall temperature range is necessary to achieve an accuracy of 93.6% even for previously unknown damage locations.

### 3.5. Comparison to a State-of-the-Art Neural Network

Since neural networks (NN) are nowadays often used for SHM applications [39,40,41], we benchmarked our approach against a neural network approach reported for the same dataset [9]. In this study, Mariani et al. first tested several deep learning algorithms, namely, a multilayer perceptron, a recurrent neural network with long short-term memory, and a WaveNet-based causal dilated convolutional neural network (CNN), on a reference guided wave SHM dataset using a threshold-based OBS + BSS as the benchmark. They found that multilayer perceptrons and recurrent neural networks were not able to significantly outperform OBS + BSS, whereas the causal dilated CNN delivered high accuracy within reasonable training time and was therefore applied to the experimental guided wave dataset for varying temperature [12]. Mariani et al. achieved 100% accuracy on the testing data for the transducer combination T_4_ to T_10_ with a high-pass filter (Butterworth), down sampling (factor 6), and BSS (undamaged plate at 40 °C) as pre-processing. A more detailed description as well as the architecture of the causal dilated CNN can be found in the original paper [9].

To compare our approach with these results for the causal dilated CNN, we also evaluated the transducer combination T_4_ and T_10_ for model building and replicated the grouping of Mariani et al. for training, validation, and testing data. Thus, training data contained D_16_, D_24_, and 50% of UG_2_; validation data contained D_12_ and 25% of UG_2_; and testing data contain D_04_ and 25% of UG_2_. The split of UG_2_ into the corresponding groups was based on a training–validation–training–testing pattern with a 1.5 °C step size (e.g., data from 20 °C–21.5 °C were used for training, 22 °C–23.5 °C for validation, 24 °C–25.5 °C for training, 26 °C–27.5 °C for testing, 28 °C–29.5 °C again for training, etc.).

**Figure A2:**
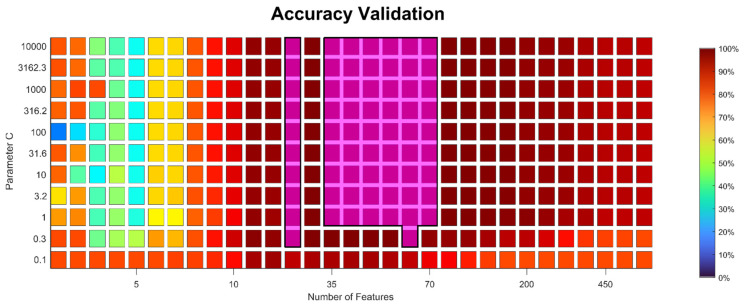
Resulting accuracy of the validation data for various parameter combinations (parameter C of the SVM and number of features selected by RELIEFF). Parameter combinations within the purple boxes achieve 100% validation accuracy. The gap between the two purple boxes consists of parameter combinations achieving an accuracy of 99.5%.

The model was built using the improved approach described above, with BFC as a feature extractor, PCC for feature pre-selection, RELIEFF for the final feature selection, and SVM with RBF kernel as a classifier. Out of the possible combinations for the hyper-parameters, the algorithm selected 30 as the best number of features and 10,000 as the value for parameter C. Actually, a wide range of hyper-parameter combinations achieved a validation accuracy of 100%, showing that the approach is robust (Appendix C, Figure A2). After hyper-parameter selection and before applying the model on the test data, it was again trained with all training and validation data. The achieved prediction accuracy of 100% for damage D_04_ matches the result reported by Mariani et al.

The computational time for our model was 185 s on an Intel^®^ Core™ i7 8650U CPU, which is also similar to the 5 min training time for the causal dilated CNN reported by Mariani et al. using one NVIDIA^®^ Quadro RTX™ 6000 GPU (2000 epochs). Note, however, that the CPU used in our study only has a theoretical computational performance of 0.442 TFLOPS (tera floating-point operations per second) compared to 16.3 TFLOPS of the GPU.

At first glance it might seem that the causal dilated CNN required less data pre-processing. However, hyper-parameter optimisation (HPO) is not described by Mariani et al. in their study. It is well known that HPO of NN models often requires significant (hardware and human) resources. Over the last few years, different approaches [42,43,44] have been proposed to solve this problem. Existing methods and frameworks to find a proper architecture and HPO of NNs are often computationally expensive and/or application-specific [43,44]. On the other hand, HPO for our proposed approach is simple and clear, as demonstrated by Figure A2 (Appendix C), which is one of the advantages of using classical ML methods (feature extraction/feature selection/simple classification) instead of deep NN models. Furthermore, our approach directly provides relevant features, i.e., a physically interpretable result, whereas NN models are often a black box and require significant additional effort to allow for interpretation. 

## 4. Conclusions

This paper presents results of an automated ML framework applied to damage detection for guided wave-based structural health monitoring. We demonstrate that damage locations were correctly classified with a success rate of 88% without domain-specific knowledge or hyper-parameter tuning. By interpreting the results of the automated toolbox and a slight tuning of the hyper-parameters, an accuracy of 96.2% was achieved using a realistic group-based validation scenario while keeping the improvement time and effort low and, more importantly, achieving physically interpretable results. 

Due to the small dataset size (for a single transducer combination T4 to T10 at 40 kHz excitation frequency) with the unbalanced ratio between the number of measurements for damaged and undamaged structures, plus the lab setup with reduced ambient influences, no conclusion can be drawn regarding how well the approach would perform in real-life applications. Edge reflections, boundary conditions, and complex geometries might lead to lower performance.

Therefore, application of the presented ML framework on real damages and CFRP components in extended temperature ranges (e.g., −50 °C to +100 °C), as well as the influence of the distance between sensors and damages, edge effects, and other damage types, offer an interesting field for future research.

## Figures and Tables

**Table 1:** Position of the transducers and the damage locations [12]. The distance of the damage locations to the direct signal path had been calculated.

Label	Position on x-Axis (mm)	Position on y-Axis (mm)	Distance to Signal Path (mm)
Transducer positions			
Transducer 4	210	470	0
Transducer 9	290	30	0
Damage positions			
Damage 04	65	400	155
Damage 12	195	330	40
Damage 16	335	260	85
Damage 24	450	190	186

**Table 2:** Feature extraction and selection methods of the automated toolbox [17].

Methods	Abbreviation	Literature
**Feature Extraction Methods**		
Adaptive linear approximation	ALA	[18]
Principal component analysis	PCA	[19]
Best Fourier coefficients	BFC	[20]
Best Daubechies wavelets	BDW	[21]
Statistical moments	SM	[22]
**Feature Selection Methods**		
Recursive feature elimination support vector machines *	RFE-SVM	[23,24]
RELIEFF *	RELIEFF	[25,26]
Pearson correlation coefficient	PCC	[27]

* Before this feature selection method is applied, the number of features is reduced to 500 in a first feature selection step based on the Pearson correlation coefficient.

**Table 3:** Parameters and values used for the grid search approach to improve the ML model. “Number of features” means the selected features that are used for classification. Bold numbers indicate the selected hyper-parameters for Section 3.5.

Hyper-Parameter	# of Values	Values
Number of features	31	1, 2, …, 10, 15, 20, …, **25**, …, 50, 60, 70, …, 100, 150, …, 500
Regularisation parameter *C*	11	0.1, **0.3**, 1, 3.2, 10, 31.6, 100, 316.2, 1000, 3162.3, 10,000

**Table 4:** Overview of the testing accuracies of all 15 combinations of the automated toolbox, derived in a previous study [36]. The highest testing accuracy is shown in bold.

Testing Accuracy for Each Algorithm Combination of the Automated Toolbox					
	PCA	BFC	BDW	ALA	SM
Pearson	42%	73%	42%	31%	81%
RELIEFF	42%	80%	43%	31%	78%
RFE-SVM	52%	**88%**	48%	31%	81%

**Table 5:** Overview of the testing accuracy and number of misclassifications of the improved algorithms (BFC, RELIEFF with Pearson pre-selection, RFE-SVM) of the toolbox for GW-based SHM.

Results of the Improved Algorithms of the Toolbox							
Damage Case	UG_1_	UG_2_	D_04_	D_12_	D_16_	D_24_	Total
Number of samples	80	161	161	161	161	161	885
Misclassifications	1	3	0	0	0	29	33
Accuracy	98.7%	98.1%	100%	100%	100%	82.0%	96.2%

**Table 6:** Ranked BFC features, i.e., frequencies, for transducer combinations 4 and 9 with their rank, total selections, amplitude selections, and phase selections. Ranking is based on how often the respective frequency is selected either as an amplitude or a phase feature in the six different LOGOCV models. Four frequencies are selected six times each.

	Ranked Frequencies (BFC Features)				
Nr.	Rank	Frequency	Total Selections	Amplitude Selections	Phase Selections
1	1	38.9 kHz	10	4	6
2	2	42.7 kHz	9	6	3
3	3	45.0 kHz	8	3	5
4	4	35.9 kHz	7	2	5
5	5	27.5 kHz	6	6	0
6	5	36.6 kHz	6	5	1
7	5	42.0 kHz	6	0	6
8	5	45.8 kHz	6	3	3

**Table 7:** Position of transducers 1 and 7.

Label	Position on x-Axis (mm)	Position on y-Axis (mm)
Transducer 1	450	470
Transducer 7	450	30

**Table 8:** Distance of the damage locations from the signal path between transducers 1 and 7.

Label	Distance from Signal Path (mm)
Damage 04	385
Damage 12	255
Damage 16	11.5
Damage 24	0

**Table 9:** Accuracy and number of misclassifications of the improved algorithm (BFC for feature extraction, RELIEFF for feature selection, SVM with RBF kernel for classification validated with LOGOCV) for the combination of transducers 1 (sender) and 7 (receiver).

Validation Results of the Improved Algorithm for the Combination of Transducers 1 and 7							
Damage case	UG_1_	UG_2_	D_04_	D_12_	D_16_	D_24_	Total
Misclassifications	4	39	133	68	0	0	244
Accuracy	94.9%	75.8%	17.4%	57.7%	100%	100%	72%

**Table 10:** Resulting testing accuracy over temperature extrapolation. The extrapolated temperatures were not used for the model building and only used for testing.

Resulting Testing Accuracy for a Certain Temperature Extrapolation							
Temperature extrapolation	2 °C	4 °C	6 °C	8 °C	10 °C	12 °C	14 °C
Testing accuracy	100%	100%	100%	97.0%	96.8%	93.6%	83.7%

## Data Availability

The data “Guided wave data for varying temperature” presented in this study are openly available in the Open Guided Waves Platform at https://doi.org/10.6084/m9.figshare.9863465 [12].

## Supplementary Materials

The Automated ML Toolbox for Cyclic Sensor Data can be downloaded at: https://github.com/ZeMA-gGmbH/LMT-ML-Toolbox (accessed on 28 December 2021); The Automated ML Toolbox DAV³E can be downloaded at: https://www.lmt.uni-saarland.de/index.php/de/forschung/157-dav3e (accessed on 28 December 2021).

## Abbreviations

The following abbreviations are used in this manuscript:

ALA	Adaptive linear approximation
BDW	Best Daubechies wavelets
BFC	Best Fourier coefficients
BSS	Baseline signal stretch
CFRP	Carbon fibre-reinforced plastic
CNN	Convolutional neural network
CPU	Central processing unit
CV	Cross-validation
D_XX_	Damage number XX
DB	Database
FE	Feature extraction
FS	Feature selection
GPU	Graphics-processing unit
GW	Guided waves
HPO	Hyper-parameter optimisation
LDA	Linear discriminant analysis
LOGOCV	Leave-one-group-out cross-validation
ML	Machine learning
NN	Neural network
OBS	Optimal baseline selection
PC	Principle component
PCA	Principle component analysis
PCC	Pearson correlation coefficient
RBF-Kernel	Radial basis function kernel
RFE-SVM	Recursive feature elimination support vector machines*
RH	Relative humidity
RMSE	Root mean square error
SHM	Structural health monitoring
SM	Statistical moments
SVM	Support vector machines
T_X_	Transducer number X
TFLOPS	Tera floating-point operations per second
UG	Undamaged group

## Appendix B

The following section describes the mathematical background of the applied ML algorithms (BFC, PCC, RELIEFF, RFE-SVM).

First, from the pre-processed signal with 13,108 samples per measurement, the frequency domain representation is calculated by using a discrete Fourier transform (A1) as well as the corresponding phase angles. Here, the standard implementations *fft()* and *phase()* of MATLAB 2021a are used [45,46]. It holds

(A1) $$Y\left(k\right)=\overset{n}{\underset{j=1}{\sum }}X\left(j\right)W_{n}^{\left(j-1\right)\left(k-1\right)}\;with\;W_{n}=e^{-2\pi i/n},$$

where Y(k) denotes the Fourier transform of the input signal X with length n and the imaginary unit i.

The resulting two-sided spectrum is converted into a single-sided amplitude spectrum. All necessary steps can be found in [45]. Next, the computed frequencies are ranked according to the absolute value of their amplitudes, and the highest 10% (1310 amplitudes) with their corresponding phase angle (1310 angles) are used as features (2620 features).

For the first feature (pre-)selection step with Pearson linear correlation coefficient r down to 500 features, it holds

(A2) $$r\left(a,b\right)=\frac{\sum_{i=1}^{n}\left(X_{a,i}-{\bar{X}}_{a}\right)\left(Y_{b,i}-{\bar{Y}}_{b}\right)}{\sqrt{\left\{\sum_{i=1}^{n}{\left(X_{a,i}-{\bar{X}}_{a}\right)}^{2}{\left(Y_{b,i}-{\bar{Y}}_{b}\right)}^{2}\right\}}}\;\mathrm{with}\;\bar{X_{a}}=\frac{1}{n}\sum_{i=1}^{n}\left(X_{a,i}\right),\;\mathrm{and}\;\bar{Y_{b}}=\frac{1}{n}\sum_{j=1}^{n}\left(X_{b,j}\right),$$

where X denotes the matrix of pre-selected features and Y the target. $X_{a}\in {\mathbb{R}}^{n\times 1}$ represents a column of matrix X and $Y_{b}\in {\mathbb{R}}^{n\times 1}$ a column of matrix Y.

Before applying RELIEFF as main feature selection method, the preselected features get standardised. RELIEFF is implemented in MATLAB by using the built-in *knnsearch()* function to determine the indexes of the three nearest neighbours (city block distance metric) of the same group (hits), and the nearest neighbours of the other groups (misses) [47]. The features are eventually ranked, with the features with a high distance to other groups (misses) and low distance to the same group (hits) achieving a higher ranking. Another internal 10-fold CV determines the necessary number of selected features.

The classifier support vector machine with radial basis function kernel (RBF kernel) tries to find a multidimensional hyperplane

(A3) $$\vec{w},\vec{x}+b=0\;,$$

with $\vec{w}$ being a normal vector and b the bias term to optimally separate two classes [32]. The goal of training an L1-norm SVM is to maximise the generalisability of the model towards untrained data by minimising

(A4) $$Q\left(\vec{w},b,\vec{\xi }\right)=\frac{1}{2}{\left|\vec{w}\right|}^{2}+C\overset{M}{\underset{i=1}{\sum }}\xi_{i},$$

as shown in [31].

Misclassifications need to be tolerated but kept track of using the parameter $\vec{\xi },$ where C acts as a regularisation parameter. Depending on which side of this hyperplane new datapoints appear on, they are classified as either class one or class two. To also separate data that show non-linear behaviour, the so-called kernel trick transforms the data into a higher dimensional feature space, in which the hyperplane might be able to linearly separate the two classes. The chosen RBF kernel (5) transforms data into an infinite-dimensional feature space. Here, every support vector is the centre point of a radial Gaussian function

(A5) $$K\left(\vec{x},{\vec{x}}^{\text{′}}\right)=\exp\left(-\frac{\left|\vec{x}-{\vec{x}}^{\text{′}}\right|}{2\sigma }\right)$$

where σ corresponds to the radius of the Gaussian function. Note that the parameter σ is automatically optimised in an heuristic procedure by the MATLAB function *fitcecoc()* [48] while using *templateSVM()* [49] with *KernelScale* set to *auto*. To ensure reproducibility, a seed (*default*, respectively 0) is specified for the random number generator of MATLAB. This results in the following optimization problem [31,32]:

(A6) $$\mathrm{maximise}\;Q\left(\alpha \right)=\overset{M}{\underset{i=1}{\sum }}\alpha_{i}-\frac{1}{2}\overset{M}{\underset{i,j=1}{\sum }}\alpha_{i}\alpha_{j}y_{i}y_{j}K\left({\vec{x}}_{i},{\vec{x}}_{j}\right)\;,$$

where *M* denotes the number of α non-negative Lagrange Multiplicators, y the class, and $K\left({\vec{x}}_{i},{\vec{x}}_{j}\right)$ the kernel function. Once the SVM is trained, new data can be classified by using

(A7) $$D\left(\vec{x}\right)=\underset{i\in S}{\sum }\alpha_{i}y_{i}K\left(\vec{x_{i}},\vec{x_{j}}\right)+b\;\mathrm{is}\;\mathrm{classified}\;\mathrm{into}\;\{\begin{array}{c} \mathrm{Class}\;1,\;if\;D\left(\vec{x}\right)>0\\ \mathrm{Class}\;2,\;if\;D\left(\vec{x}\right)<0 \end{array},$$

where S denotes the set of support vector indices. Strategies for handling multiclass classification problems can be found in [31].
